# The problem of defecation disorders in children is underestimated and easily goes unrecognized: a cross-sectional study

**DOI:** 10.1007/s00431-018-3243-6

**Published:** 2018-09-27

**Authors:** Marjolijn E. W. Timmerman, Monika Trzpis, Paul M. A. Broens

**Affiliations:** 1 Department of Surgery, Division of Pediatric Surgery, University of Groningen, University Medical Center Groningen, P.O. Box 30 001, 9700 RB Groningen, the Netherlands; 2 Department of Surgery, Anorectal Physiology Laboratory, University of Groningen, University Medical Center Groningen, Groningen, the Netherlands

**Keywords:** Constipation, Fecal incontinence, Help-seeking behavior, Prevalence, Stool frequency, Stool consistency

## Abstract

We aimed to study constipation and fecal incontinence in terms of prevalence, recognizing the disorders, help-seeking behavior, and associated symptoms. In this cross-sectional study, 240 children (8 to 18 years) from the general Dutch population completed a questionnaire about defecation disorders. After exclusions for anorectal/pelvic surgery or comorbidities, we analyzed 212 children. The prevalence of constipation was 15.6%; in a quarter of the cases, it co-occurred with fecal incontinence. We found 3% fecal incontinence without constipation. Even though children with a defecation disorder rated their bowel habits worse compared to children without defecation disorders (*P* < 0.001), 46% constipated children and 67% fecally incontinent children rated their bowel habits as good or very good. Moreover, 21 to 50% of children with a defecation disorder did not mention their symptoms to anybody. Interestingly, most constipated children had “normal” stool frequencies (64%) and consistencies (49%).

*Conclusion:* The prevalence of constipation and fecal incontinence is quite high in children. Stool frequency and consistency is normal in half the constipated children, which may complicate the recognition of constipation. Finally, a considerable number of children does not recognize their disorders as constituting a problem and does not seek help, which leads to an underestimation of these disorders.

**Table:** 

**What is Known:** • *Constipation and fecal incontinence are common in children, but their prevalence rates may be underestimated due to a variety of reasons.* • *Diagnosing these disorders remains challenging owing to the variety of symptoms and co-existence with other diseases.*	
**What is New:** • *The prevalence of constipation and fecal incontinence in children is high.* • *Many children do not recognize their defecation disorders as constituting a problem and do not seek help, which leads to an underestimation of the problem of these disorders.*

**Electronic supplementary material:**

The online version of this article (10.1007/s00431-018-3243-6) contains supplementary material, which is available to authorized users.

## Introduction

Constipation and fecal incontinence are commonly found in children [20, 23]. Their impact is considerable (also considering that constipation can result in recurrent abdominal pain), not only on the quality of life of the children concerned and their families [5, 9, 21], but also on the health care system [16, 20]. In addition, constipation can also result in recurrent abdominal pain [6]. The prevalence rates of these defecation disorders vary widely across populations. In children the rates of constipation vary from 1 to 30%, and the rates of fecal incontinence vary from 1.6 to 4.4% [2, 8, 14, 25]. These large differences in prevalence rates can be explained partly by differences in demographic characteristics, dietary patterns, and respondent characteristics, such as age, sex, and body mass index, of the populations studied [8, 17, 18, 28]. The method of inclusion and diagnosis can also influence prevalence rates [4, 26]. Moreover, the extent to which children or their parents recognize the existence of a defecation problem might influence help-seeking behavior, the percentage of doctor’s visits, and consequently might influence the reported prevalence rates of defecation disorders. Furthermore, the prevalence of constipation and fecal incontinence can vary considerably between children who suffer from an underlying medical disorder, such as Hirschprung’s disease or congenital anorectal malformations, and children without any known medical disorder [18]. Many studies focused on investigating constipation and fecal incontinence in the setting of the general practitioner or in a hospital [2, 8]. However, based on our clinical experience, we think that mostly children with frequent symptoms and not with sporadic symptoms of constipation or fecal incontinence visit a general practitioner or medical specialist. This may be due to the fact that frequently appearing symptoms influence the quality of life more severely than sporadically appearing fecal problems [5, 9, 21]. Consequently, prevalence rates as reported in hospital setting might be biased and yield an underestimation of the prevalence in the total population.

Therefore, our first objective was to study the prevalence of constipation and fecal incontinence in the general Dutch pediatric population. Our second objective was to investigate factors that can influence the prevalence rate of these defecation disorders, namely if the disorders are recognized by children and/or their parents, if children seek help, and if key symptoms occur. From our previous study, we know that constipation and fecal incontinence are still a taboo and people do not openly talk about them [11]. To obtain detailed information about fecal problems of children in the Netherlands, we decided to perform anonymous cross-sectional study. For this, we used the pediatric version of the Groningen Defecation and Fecal Continence (DeFeC) questionnaire of which the feasibility, reproducibility, and validity of this questionnaire have been tested in the adult population [12].

## Material and methods

This cross-sectional study was performed in the Dutch population. It was conducted in compliance with requirements of our local Medical Ethics Review Board (Medical Ethical Committee of the University Medical Center Groningen). Between September and December 2015, respondents were randomly selected from an already existing database of inhabitants throughout the Netherlands by the external company Survey Sampling International in Rotterdam, the Netherlands. The selected respondents were approached by the company and asked to fill in the Groningen Pediatric Defecation and Fecal Continence questionnaire (provided in supplementary file) [12]. Participants between the ages of 8 and 18 years old were approached through their parents and invited to participate in this study, in accordance with the Dutch law. Parental and/or informed consent was obtained for all participants. Inclusion was continued until a predetermined number (based on age and gender, according to Dutch demographics) of completed questionnaires per demographic group had been obtained. The company paid 0.30 euro per completed questionnaire.

### Analysis of respondents

Altogether 1035 children who met the abovementioned selection criteria were approached to participate in the study. Out of this group, 241 children and their parents agreed to participate and completed the questionnaire eventually with the help of their parents (response rate of 23%). After a data quality check by Survey Sampling International, one participant was excluded due to illogic answers. Furthermore, in order to follow the Rome IV criteria for functional disorders, we excluded 28 children because they had a history of anorectal or pelvic surgery, a diagnosed comorbidity, or used medication for their comorbidities that could influence bowel habits. Excluded types of surgery were partial bowel resection with anastomosis (*n* = 1), surgery for perianal fistula (*n* = 1), surgery for hemorrhoids (*n* = 1), surgery for sacrococcygeal teratoma (*n* = 1), and appendectomy (*n* = 2). Furthermore, excluded comorbidities were inflammatory bowel disease (*n* = 1), irritable bowel syndrome (*n* = 7), diabetes mellitus (*n* = 2), slow transit constipation (*n* = 1), spina bifida (*n* = 1), juvenile rheumatoid arthritis (*n* = 1), gastroesophageal reflux disease (*n* = 1), and Ollier disease (*n* = 1). Respondents who used the following medications were also excluded: amitriptyline, antihistamines, dexamphetamine, esomeprazole, folic acid, inhalation glucocorticoids, liraglutide, lorazepam, melatonin, metformin, methotrexate, methylphenidate, mometason, nonsteroidal anti-inflammatory drug, omeprazole, ondansetron, oxycodone, paroxetine, pramipexole, quetiapine, sertraline, temazepam. We finally included 212 children for analysis.

### Criteria for constipation and fecal incontinence

The Groningen Pediatric Defecation and Fecal Continence questionnaire consists of multiple validated scores for constipation and fecal incontinence [12]. For this study, we analyzed children from the general Dutch population who had not all voluntarily reported their possible symptoms to a medical specialist. We were therefore of the opinion that it was neither ethical nor feasible to subject them to the digital rectal examination or additional investigations that are required for applying the pediatric Rome IV criteria for constipation and fecal incontinence [7]. For the purpose of this study, we used the adult Rome IV criteria that do not require physical examination or additional investigations. Consequently, for constipation, we used the following criteria: straining, lumpy or hard stools (Bristol stool form scales 1 and 2), incomplete evacuation, anorectal blockage, manual maneuvers to facilitate defecation, and reduced stool frequency (less than 3 bowel movements per week). We simplified the questions on straining, obstruction, or incomplete evacuation by not asking if it occurred at least 25% of the defecations, because we thought this would be too difficult to answer for children. We dichotomized the answers of the abovementioned question, namely symptoms that occurred at least several times a month were classified as one (i.e., symptoms present) and symptoms that occurred less frequently were classified as zero (i.e., no symptoms present). If respondents had at least two of the aforementioned symptoms, also rarely had loose stools without using laxatives, and had insufficient criteria for irritable bowel syndrome, they fulfilled the criteria for constipation [10]. The adult Rome IV criteria were also used for irritable bowel syndrome [10]. For fecal incontinence, the following criteria were used: uncontrolled passage of fecal material in an individual with a developmental age of at least 4 years for the last 3 months [22].

### Statistical analysis

SPSS 23.0 for Windows (IBM SPSS Statistics, IBM Corporation, Armonk, NY) was used for the statistical analysis of the data. A descriptive analysis was performed for all variables. Normally distributed, continuous data were described as means and standard deviations, and analyzed with an independent sample *t* test. Categorical data were described as numbers and proportions and analyzed with a chi-square test or Fisher exact test. Regression analysis was used to correct for possible confounding factors. We considered *P* values below 0.05 as statistically significant.

## Results

### Respondent characteristics

Of the 1035 children who were approached, 240 children agreed to participate and were included (response rate of 23%). Of these children, 28 were excluded due to a history of surgery or comorbidities. The respondent characteristics of the 212 analyzed children, including age, sex, body mass index, living environment, and medication use, are described in Table 1.

**Table 1:** Respondent characteristics

	Analyzed respondents *N* = 212
Age, mean (SD)	13.1 (2.81)
Sex, *n* (%)	
Boys Girls	119 (56) 93 (44)
Body mass index category*, *n* (%)	
Underweight Normal weight Overweight Obese	17 (8) 145 (69) 23 (11) 24 (12)
Living environment, *n* (%)	
Rural Urban	85 (40) 127 (60)
Use of medication for bowel disorders, *n* (%)	
No Laxatives Enemas Antidiarrheals	194 (95) 7 (3) 1 (0.5) 1 (0.5)
Use of medicine for comorbidities, *n* (%)	
No Yes	210 (99) 2 (1)**

*Variable contains missing data. **These patients only used oral contraceptives

### Prevalence of defecation disorders

The prevalence of constipation was 15.6% in our study group, a quarter of whom also had fecal incontinence symptoms. Most of the constipated children (61%) had symptoms of straining, obstruction, or sensation of incomplete defecation daily or several times a week, while 39% of the constipated children had these symptoms several times a month. The criteria for fecal incontinence without having constipation were met by 3% of our study group. Most of the fecal incontinent children (67%) had symptoms several times a month, while 33% had symptoms several times a week. Age and sex had no significant influence on the prevalence rates of the defecation disorders.

### Self-estimation of the quality of bowel habits

**Fig. 1:**
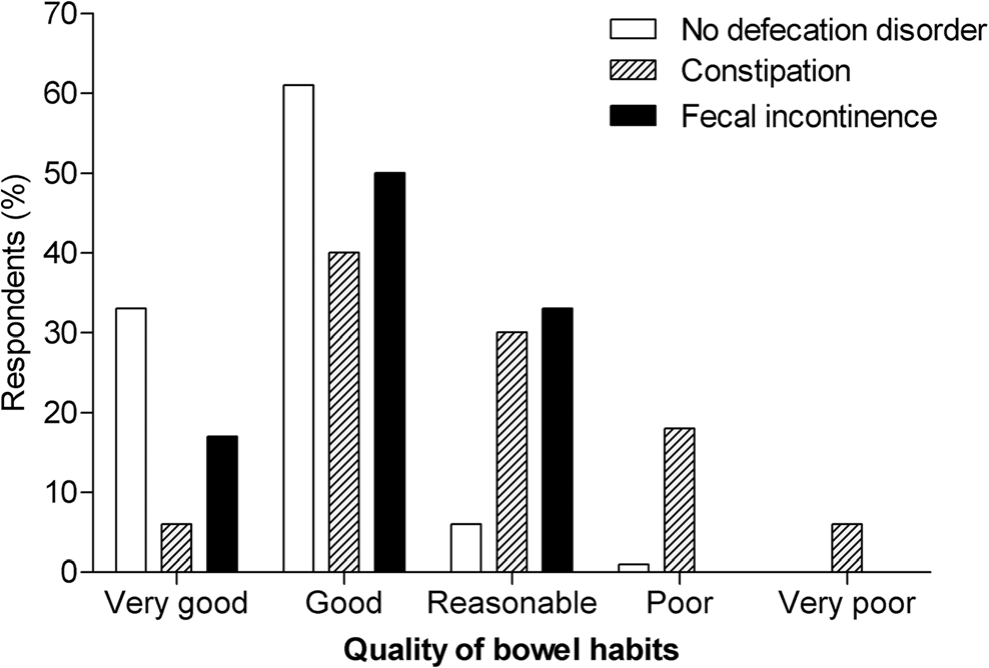
Self-estimation of the quality of bowel habits. Self-estimation of quality of bowel habits given by children with and without different defecation disorders. *P* < 0.001 for constipation and fecal incontinence compared to no defecation disorder

We analyzed how children with constipation or fecal incontinence qualified their bowel habits in comparison with children without a defecation disorder (no constipation, fecal incontinence, or irritable bowel syndrome). We found that 46% of children with constipation rated the quality of their bowel habits as “good” or “very good,” compared to 93% of the children without any defecation disorder (*P* < 0.001) (Fig. 1). The way constipated children, who also experienced fecal incontinence symptoms, rated their bowel habits was similar to children who only had constipation. In case of fecal incontinence, 67% of the children considered their bowel habits as “good” or “very good” compared to the 93% of the children without any defecation disorder (*P* < 0.001). There was no significant difference in how children with constipation rated the quality of their bowel habits compared to children with only fecal incontinence (*P* = 0.23).

### Help-seeking behavior

**Fig. 2:**
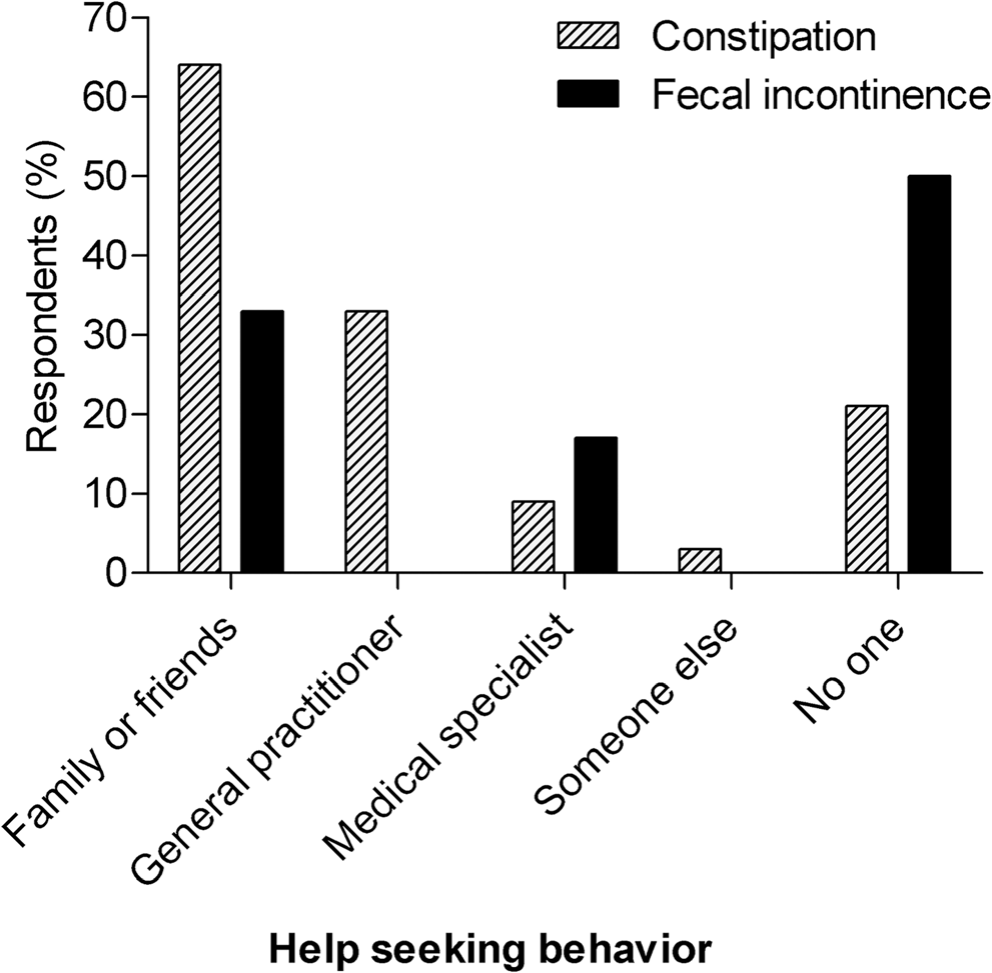
Help-seeking behavior. Help-seeking behavior of children with constipation and fecal incontinence

We assessed help-seeking behavior of children with constipation or fecal incontinence by asking if they had ever mentioned their defecation problems with anybody, and if they had, whom had they spoken to (Fig. 2). Most constipated children spoke about their symptoms with family and friends (64%) or with their general practitioner (33%). In addition, 21% of the constipated children did not mention their defecation problems to anybody, of which half had symptoms daily or several times a week. The pattern of help-seeking behavior was similar in constipated children who also had fecal incontinence symptoms in comparison to constipated children without fecal incontinence. In case of fecal incontinence, 50% of the children, who experienced symptoms several times a month, did not mention their accidental loss of stool to anyone. But if they did, they mostly spoke about it to family and friends (33%), instead of speaking to a general practitioner (0%) or a medical specialist (17%).

### Stool frequency and consistency

**Fig. 3:**
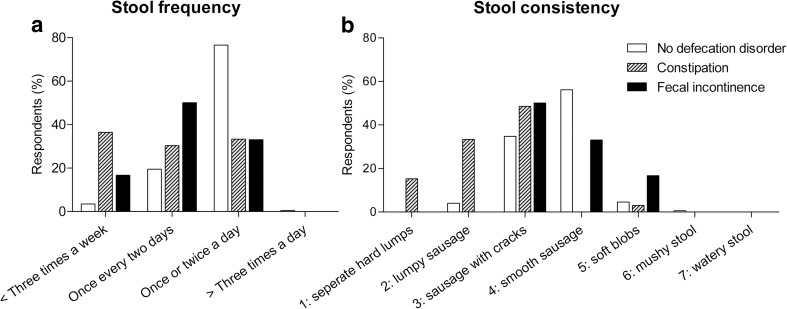
Stool frequency and consistency. **a** Stool frequency. *P* < 0.001 for constipation and fecal incontinence compared to no defecation disorder. **b** Stool consistency. *P* < 0.001 for constipation and fecal incontinence compared to no defecation disorder

Finally, we studied associated symptoms of constipation and fecal incontinence by comparing children’s stool frequency and stool consistency to those of children without a defecation disorder (without constipation, fecal incontinence, or irritable bowel syndrome) (Fig. 3). Children with constipation had a significantly lower stool frequency and a more solid stool consistency compared to children without a defecation disorder (*P* < 0.001). Nevertheless, 64% of the children with constipation had normal stool frequencies (defined as “once every two days” or “once to twice a day”), while 49% of the children had a normal stool consistency (defined as Bristol Stool Chart 3 or 4: sausage with cracks or smooth sausage). Stool frequencies and consistencies of constipated children who also reported symptoms of fecal incontinence were similar to those of children who only had constipation. Furthermore, the stool frequency and consistency of fecal incontinent children was not significantly different compared to children without a defecation disorder (*P* = 0.44). Most children with fecal incontinence had normal stool frequencies and consistencies (83%).

## Discussion

We demonstrated that in our study group of the Dutch pediatric population, 15.6% had constipation, a quarter of whom also had fecal incontinence. Furthermore, 3% of our study group had fecal incontinence without underlying constipation. In addition, we observed the prevalence rates of constipation and fecal incontinence in children, without excluding those with anorectal/pelvic surgery or comorbidities, to gain insight into the true magnitude of defecation disorders in the general population (data not shown). These prevalence rates, however, were similar to the rates of children without comorbidities. Thus, the prevalence rates of constipation and fecal incontinence in our study are quite high, but seem comparable to those reported by other studies [2, 8, 25]. Nevertheless, it is important to note that for ethical and practical reasons, we used the adult Rome IV criteria instead of the pediatric criteria, as a result of which our prevalence rates may deviate from other studies. Furthermore, our prevalence rates may also differ from others studies because we analyzed the general population and not a subpopulation of children seen by general practitioners or medical specialists. Therefore children with a defecation disorder who did not seek help were also included in the prevalence rates. This conclusion is supported by our observation that approximately one-fifth to half of the children who had constipation and/or fecal incontinence did not mention their problems to anyone, which is probably due to the taboo on talking about defecation problems as suggested by another study [19]. This leads to an underestimation and involuntary ignorance of the scale of these problems.

Furthermore, we found that even though children with constipation and fecal incontinence rated the quality of their bowel habits significantly lower than children without a defecation disorder, around half of the children with constipation or fecal incontinence still rated their bowel habits as good or very good. This implies that children do not always see their defecation disorders as a problem, which in turn may lead to less help-seeking behavior for their complaints [13, 15, 27]. Thus, poor recognition of actually having a defecation problem by patients together with the taboo on talking about defecation disorders may lead to underestimating the prevalence of constipation and fecal incontinence in children.

Finally, the prevalence of defecation disorders can also be underestimated because doctors fail to recognize the problem. Many children with constipation had a normal stool frequency (64%) and consistency (49%), as has also been found by others [1, 24]. We can therefore conclude that merely questioning children about their stool frequency and consistency is insufficient when screening for constipation. Thus, medical specialists should interview children who are suspected of constipation extensively and they should, for example, also ask about straining, incomplete evacuation, and anorectal blockage [7, 10, 26].

Some limitations of our study need to be addressed. It was not possible to use the pediatric Rome IV criteria for functional constipation and fecal incontinence to analyze the prevalence rate of defecation disorders in the general population with a questionnaire, because these criteria require physical and additional examinations. We considered this both unethical and unfeasible. For the purpose of this study, we therefore used the adult Rome IV criteria for constipation and fecal incontinence and simplified the questions to make them understandable for children. Consequently, our prevalence rates may differ from other studies. Furthermore, the response rate was relatively low, probably due to the taboo on talking about defecation. Such a taboo is unfortunately still present in many countries, also in the Netherlands [11]. A selection bias towards collecting relatively many children with defecation complaints may also have occurred, because people with complaints are possibly more likely to fill out questionnaires related to their problems [3]. Due to the relatively small sample size, it was not possible to perform more detailed analyses on the recognition and help-seeking behavior by for instance taking into account the frequency of symptoms. But, since the Pediatric Defecation and Fecal Continence questionnaire has been translated into English, this study can easily be repeated in other countries in the future, which will contribute to the generalizability of the results. In future studies, this questionnaire could also be used to study constipation and fecal incontinence in more depth in the general practitioner or hospital setting. In addition, it would be interesting to further investigate the reasons why children do not talk about their disorders.

## Conclusion

The prevalence of constipation and fecal incontinence is quite high in our study of the general Dutch pediatric population. Furthermore, a large proportion of children with a defecation disorder does not recognize it as a problem and does not seek help. This can lead to underestimating the prevalence of these disorders. Finally, most constipated children report normal stool frequencies and consistencies, which may contribute to problems regarding recognition if physicians limit their screening for constipation to these criteria alone.

## Electronic supplementary material

ESM 1 (DOCX 1044 kb)
