# Supplementary material for: The problem of defecation disorders in children is underestimated and easily goes unrecognized: a cross-sectional study
Source: Eur J Pediatr. 2018 Sep 27;178(1):33–9. doi: 10.1007/s00431-018-3243-6 (PMC6311181; doi:10.1007/s00431-018-3243-6)
Supplement: Supplementary file 1 — (DOCX 1044 kb) [file 431_2018_3243_MOESM1_ESM.docx]

**The Groningen Pediatric Defecation & Fecal Continence Questionnaire**

Instructions:

1. Answer the questions by ticking the box next to your answer. Please tick just one answer to each question (unless you are invited to give more than one answer).

2. Although some of the questions may seem very similar, each one gives us important information. Some of the questions might be about problems you do not have, but we would like to know this too. Please answer every question (unless you are specifically told to go to another question).

3. There are no right or wrong answers. If you are unsure about how to answer a question, try to choose the answer that seems best.

4. If you have any comments about the questionnaire, or if there is anything else you would like to say but which has not been covered by the questions, you can add your own comments at the end of the questionnaire.

5. Your answers will be treated in the strictest confidence.

**Personal details**

Surname ____________________

First name ____________________

Date of birth ____________________

Height (cm) ____________________

Weight (kg) ____________________

0.1 Are you a boy or a girl?

Boy

Girl

0.2 How old are you (in years)?

__________

0.3 In which province do you live?

Drenthe  Noord-Brabant

Flevoland  Noord-Holland

Friesland  Overijssel

Gelderland  Utrecht

Groningen  Zeeland

Limburg  Zuid-Holland

0.4 How big is the town or village in which you live?

I live in a village

I live in a small town with fewer than 50,000 inhabitants

I live in a medium-sized town with 50,000 to 100,000 inhabitants

I live in a large town with more than 100,000 inhabitants

0.5 In general, how would you describe your health in relation to the ability to hold and get rid of your poo?

Very good

Good

Reasonable

Poor

Very poor

**Category 1: Defecation pattern**

**The following questions are about your defecation pattern during the past six months.**

- 1. On average, how often did you go to the toilet to do a poo? (**Only tick one box**)

Less than once a month

Less than once a week

Once a week

Twice a week

Once every two days

Once or twice a day

Three to five times a day

More than five times a day

1.2 What did your poo usually look like? (**Only tick one box**)

|  | 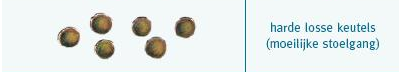 | Looks like rabbit droppings  (Separate hard lumps (hard to pass)) |
| --- | --- | --- |
|  | 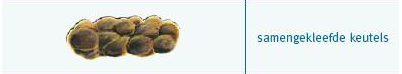 | Looks like bunch of grapes  (Sausage-shaped but lumpy) |
|  | 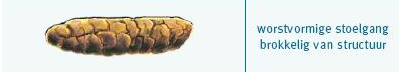 | Looks like corn on cob  (Like a sausage but with cracks on its surface) |
|  | 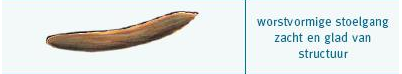 | Looks like a sausage  (Like a sausage or snake, smooth and soft) |
|  | 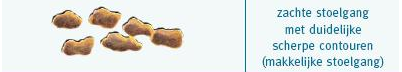 | Looks like chicken nuggets  (Soft blobs with clear-cut edges (passed easily)) |
|  | 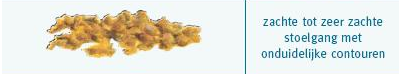 | Looks like porridge  (Fluffy pieces with ragged edges, a mushy stool) |
|  | 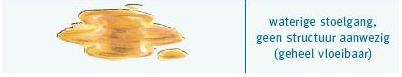 | Looks like gravy  (Watery, no solid pieces (entirely liquid)) |

**Category 2: Constipation**

**The following questions are about the problems you have had with doing a poo over the past six months.**

2.1 Did you have difficulty pooing (for example: because your poo was too hard or because you had to strain)?

Yes

No

2.1.1 If so, how long have you been having problems pooing?

0-1 year

1 to 5 years

5 to 10 years

10 to 20 years

2.2 How often did you have to strain hard to poo?

Never

Less than once a month

Several times a month

Several times a week

Every day

2.3 On average, how long did you have to strain when pooing?

Less than 5 minutes

5 to 10 minutes

10 to 20 minutes

20 to 30 minutes

Longer than 30 minutes

2.4 How often was it difficult to poo because it felt as if something was obstructing the exit?

Never

Less than once a month

Several times a month

Several times a week

Every day

2.5 How often did you feel as if you had not quite got rid of all your poo after going to the toilet?

Never

Less than once a month

Several times a month

Several times a week

Every day

2.6 How often were you unable to do a poo, despite feeling the urge (immediate need) to go to the toilet?

I was always able to

One to three times a day

Four to six times a day

Seven to nine times a day

More than nine times a day

2.7 How often did you have to return to the toilet for another poo within one hour of doing a poo?

Never

Less than once a month

Several times a month

Several times a week

Every day

2.8 How often did you have pain in your anus (bottom) while pooing?

Never

Less than once a month

Several times a month

Several times a week

Every day

2.9 Do you ever feel bloated (as if your tummy is full of air)?

Yes

No

2.9.1 If so, how badly? (You may tick more than one answer)

I only felt it myself

Other people could also see it

It made me lose my appetite or feel sick

It made me throw up

2.10 How often did you have pain or cramps in your tummy?

Never

Less than once a month

Several times a month

Several times a week

Every day

***If you did not experience pain or cramps in your tummy during the past six months, please proceed to question 3.1.***

2.10.1 If you experienced pain or cramps in your tummy, was this only while you were on your period?

No

Yes

Not applicable because I have not yet started my periods

Not applicable because I am a man

2.10.2 If you experienced pain or cramps in your tummy, did they disappear or get better after doing a poo?

Never or rarely

Sometimes

Often

Usually

Always

2.10.3 Have you had to go to the toilet to do a poo more or less frequently since the pain or cramps in your tummy started?

Yes, I go to the toilet more frequently than before

Yes, I go to the toilet less frequently than before

No, I go to the toilet just as often as before

2.10.4 Has your poo looked different since the pain or cramps in your tummy started? (Has it become harder or softer, for example?)

Yes, my poo is harder

Yes, my poo is softer

No, the consistency of my poo has not changed

**Category 3: Constipation-related questions**

**The following questions are about your diet and any remedies you might possibly have used to help you poo over the past six months.**

3.1 Do you drink at least 1.5 litres of fluids a day (10 x 150ml-cups/glasses)?

Yes

No

3.2 Do you eat at least 2 pieces of fruit a day?

Yes

No

3.3 Do you eat at least 3 tablespoons of vegetables a day?

Yes

No

3.4 Do you eat at least 3 slices of brown or wholemeal bread a day?

Yes

No

3.5 How often did you take laxatives to soften your poo/make it easier to do a poo?

Never

Less than once a month

Several times a month

Several times a week

Once a day

Several times a day

3.5.1 If you take laxatives, which one do you take and how much?

1. Medicine: _______________ How often per day: _____ Dosage: ____ ml/g

Or per week: _____

2. Medicine: _______________ How often per day: _____ Dosage: ____ ml/g

Or per week: _____

3. Medicine: _______________ How often per day: _____ Dosage: ____ ml/g

Or per week: _____

3.6 Did you follow a special diet or eat particular foods to soften your poo?

Yes, I eat /drink: ____________________

No

3.7 Did you use an enema (= injecting a small amount of a medicine into your anus (bottom) to help you poo?

Yes, medicine: ____________________ dosage: _____ ml/cc

No

3.7.1 If so, how often?

Less than once a month

Several times a month

Several times a week

Once a day

Several times a day

3.8 Did you flush your bowels with lukewarm water (injected into your bottom or with an antegrade colonic enema) to get rid of your poo?

Yes, amount: _____ ml/cc, with (if applicable): _____________

No

3.8.1 If so, how often did you flush?

Less than once a month

Several times a month

Several times a week

Once a day

Several times a day

3.9 Did you use your fingers or hands to help you poo? (You may tick more than one answer)

Yes, I press on my tummy with my hands

Yes, I use my finger to press between my buttocks, just in front of the anus (bottom hole)

Yes, I use my finger to press between my buttocks, just behind the anus (bottom hole)

Yes, I use my fingers to remove stools from my anus (bottom hole)

Yes, but in another way, namely: ____________________

No

3.9.1 If so, how often did you use your fingers or hands to help you poo?

Less than once a month

Several times a month

Several times a week

Every day

3.10 If you had difficulty pooing, did you talk to anyone about it? (You may tick more than one answer)

Not applicable, I do not have difficulty pooing

Yes, with family or friends

Yes, with my general practitioner (GP)

Yes, with a medical specialist

Yes, with someone else, namely: ____________________

No

**Category 4: Fecal continence**

**The following questions are about accidental pooing in your pants during the past six months.**

4.1 How often did you accidentally pooed a bit in your pants? (i.e. stained/soiled your underpants)

Never

Less than once a month

Several times a month

Several times a week

Once a day

Several times a day

4.1.1 If you accidentally pooed a little bit in your pants, when did this happen? (You may tick more than one answer)

When I had diarrhea

When I was desperate for the toilet

Doing a sport/ playing

For no clear reason

4.2 How often did you accidentally do a large, solid poo in your pants because you didn’t feel that you needed to go to the toilet?

Never

Less than once a month

Several times a month

Several times a week

Once a day

Several times a day

4.3 How often did you feel a strong urge (immediate need) to do a poo but were unable to reach the toilet in time?

Never

Less than once a month

Several times a month

Several times a week

Once a day

Several times a day

4.4 How often did you accidentally do a watery poo (have diarrhea) in your pants?

Never

Less than once a month

Several times a month

Several times a week

Once a day

Several times a day

4.5 How often did you accidentally pass wind?

Never

Less than once a month

Several times a month

Several times a week

Once a day

Several times a day

***If you have not accidentally done a liquid or solid poo in your pants during the past six months, please go to question 5.1.***

4.6 If you accidentally pooed in your pants, how much was this usually?

A tiny amount, about the size of a coin

Enough to make me change my underpants

Enough to make me change my underpants and trousers

4.7 If you accidentally pooed in your pants, when did this happen?

Only while I was awake

Only while I was asleep

While I was awake and while I was asleep

4.8 How often did you use panty liners or another type of pads to protect you when you accidentally pooed in your pants?

Never

Less than once a month

Several times a month

Several times a week

Once a day

Several times a day

4.9 How often did you rearrange daily routines because of accidentally pooing in your pants (e.g. you stayed at home, cancelled plans to meet friends, changed your diet)?

Never

Less than once a month

Several times a month

Several times a week

Once a day

Several times a day

4.10 Have you ever accidentally pooed in your pants shortly after doing a poo on the toilet?

Yes

No

4.11 Do you use an anti-diarrhea medicine to thicken your poo?

Never

Less than once a month

Several times a month

Several times a week

Once a day

Several times a day

4.11.1 If you use an anti-diarrhea medicine, which one do you use and how much?

1. Medicine: _______________ How often per day: ____ Dosage: ____ ml/g

Or per week: ____

2. Medicine: _______________ How often per day: ____ Dosage: ____ ml/g

Or per week: ____

3. Medicine: _______________ How often per day: ____ Dosage: ____ ml/g

Or per week: ____

4.12 Did you follow a special diet or eat particular foods to control accidental poos?

Yes, I eat/drink: ____________________

No

4.13 Did you flush your bowels with lukewarm water to stop yourself accidentally pooing?

Yes, amount: _____ ml/cc, with (if applicable): _____________

No

4.14 Have you ever talked to anyone about accidentally pooing in your pants?

(You may tick more than one answer)

Yes, with family or friends

Yes, with my general practitioner (GP)

Yes, with a medical specialist

Yes, with someone else, namely:

No

**Category 5: Urge (immediate need)**

**The following questions are about your urge (immediate need) to go the toilet over the past six months**

5.1 Did you feel the urge (immediate need) to poo before you went to the toilet?

Yes

Sometimes

No

5.2 On average, how long were you able to hold your poo in, once you had felt the urge to go to the toilet?

I was unable to hold my poo in

One minute or less (I always had to go to the toilet immediately)

Five minutes at the most

Fifteen minutes at the most

I never had to hurry

5.3 How often did you have to hurry to get to the toilet in time, to prevent yourself accidentally pooing in your pants?

Never

Less than once a month

Several times a month

Several times a week

Once a day

5.4 When you felt the urge (immediate need) to go to the toilet, could you tell the difference between wind, diarrhea and solid poo

Yes

With difficulty

No

**Category 6: Urinary incontinence**

**The following questions are about your bladder control over the past six months.**

6.1 On average, how often did you wee/pee?

Less than three times a day

Three to seven times a day

More than seven times a day

6.2 When you peed, were you able to empty your bladder in one go?

Yes, the pee stream was never interrupted

No, the pee sometimes came in bursts (stopped and started)

No, the pee always came in bursts (stopped and started)

6.3 When you peed, did you have to strain?

Yes, I always had to strain while peeing

Yes, I sometimes had to strain while peeing

No, I never had to strain while peeing

6.4 How often did you accidentally wee in your pants?

Never

About once a week or less

Two to three times a week

About once a day

Several times a day

Continuously

6.5 How much wee did you usually accidentally let out (whether you used protective pads or not)?

None

A bit (a few drops)

Quite a lot (wet underpants)

A lot (visible wet patches)

6.6 When did you accidentally wee in your pants? (You may tick more than one answer)

Never, I have never accidentally peed

Before I could reach the toilet

Whenever I sneezed or coughed

While I was asleep

Doing a sport/ playing

When I got dressed again after peeing

For no clear reason

Continuously

6.7 How often did you need to go to the toilet to pee during the night?

Never/rarely

Once or twice a week

Three to six times a week

Every night

Several times a night

6.8 How often did you feel as if you had a bladder infection in the past 6 months?

Never

Once

Several times

6.9 How often have you been treated for a bladder infection in the past 6 months?

Never

Once

Several times

**Category 8: Medical history**

**The following questions relate to conditions or operations that may affect your ability to control your poos.**

8.1 Have you ever had one of the following operations that may affect your ability to control your poos? (You may tick more than one answer)

No, I have never had an operation on my bowels/intestine, anus (bottom hole) or prostate

Removal of a section of bowel/intestine, after which the remaining parts were stitched together

Operation on a fistula in the anal cleft close to the anus (perianal fistula)

Operation on the anal sphincter

Operation for piles (haemorrhoids)

Operation on the prostate

Other, namely: ____________________

Procedure to repair a hereditary condition, such as:

Anal atresia or congenital anorectal malformation

Hirschsprung’s disease

Sacrococcygeal teratoma

8.2 Do you have (or have you had) a stoma to get rid of your poo?

Yes, a colostomy

Yes, an ileostomy

No

8.3 Do you ever have blood and/or mucus in your poo?

Yes

No

8.4 Have you ever had an injury to your anus (bottom hole), apart from an operation?

Yes, namely: ____________________

No

8.5 Have you ever had, or are you still experiencing the consequences of, one of the following illnesses? (You may tick more than one answer)

I have never had any of the illnesses listed below

Crohn’s disease or colitis ulcerosa (inflammation of the colon)

Irritable bowel syndrome

Prolapse of the rectum

Diabetes mellitus

Cerebral haemorrhage or infarction (stroke)

Another neurological conditions (e.g. paraplegia, multiple sclerosis)

Slow transit constipation

Hereditary illnesses such as:

Anal atresia or congenital anorectal malformation

Hirschsprung’s disease

Sacrococcygeal syndrome

Spina bifida

Other, namely: ____________________

8.6 Does anyone in your family also have one of the illnesses you have ticked?

Yes

No

Not applicable

8.6.1 If so, which illness has which members of your family?

Illness: ___________________ Relative: ___________________

Illness: ___________________ Relative: ___________________

Illness: ___________________ Relative: ___________________

Illness: ___________________ Relative: ___________________

8.7 Which medicines do you take at the moment (you do not need to mention the laxatives and anti-diarrhea treatments mentioned earlier)?

I do not take any other medication.

I take:

1. Medicine: How often per day: _____ Dosage _____ ml/g

___________________

1. Medicine: How often per day: _____ Dosage _____ ml/g

___________________

1. Medicine: How often per day: _____ Dosage _____ ml/g

___________________

1. Medicine: How often per day: _____ Dosage _____ ml/g

___________________

1. Medicine: How often per day: _____ Dosage _____ ml/g

___________________

1. Medicine: How often per day: _____ Dosage _____ ml/g

___________________

**This is the end of the questionnaire.**

Thank you very much for taking the time to answer these questions.

If there is anything else you would like to say, or if there is something you feel was not asked or not asked sufficiently by this questionnaire, please use the space below to leave your comments.
